# Immunoprotection Efficacy of Con A-Purified Proteins against *Haemonchus contortus* in Goats

**DOI:** 10.3390/vaccines10111891

**Published:** 2022-11-09

**Authors:** Lisha Ye, Yao Zhang, Simin Wu, Zhiheng Wang, Feng Liu, Chunqun Wang, Min Hu

**Affiliations:** State Key Laboratory of Agricultural Microbiology, College of Veterinary Medicine, Huazhong Agricultural University, Wuhan 430070, China

**Keywords:** *Haemonchus contortus*, native protein, immune protection, fecal egg count, antibody response

## Abstract

Parasitic nematodes are important pathogens that infect animals, causing significant economic losses globally. Current repeated treatments have led to widespread anthelmintic resistance in nematode populations, so vaccine development offers an alternative control approach. However, only one effective vaccine (named Barbervax) has been developed to protect animals against one of the most pathogenic nematodes of ruminants—*Haemonchus contortus* (the barber’s pole worm). This vaccine contains a dominant component, Concanavalin A (Con A) purified H11 antigen, which has been shown to induce high levels (>85%) of immune protection in sheep breeds, but in goat breeds, the immunoprotection test of this native protein is still lacking. Here, we evaluated the protective efficacy of low-dose Con A-purified proteins for controlling the *H. contortus* infection in goats. Four-month-old Boer goats were equally divided into two vaccinated groups of 5 μg and 10 μg native proteins, and one adjuvant control. Each goat was immunized subcutaneously thrice and then challenged with 7000 infective third-stage larvae (L3s). The fecal egg count (FEC), degree of anemia, antibody levels of serum and abomasum mucosa, as well as worm burdens, were detected in experimental goats. Our results showed that both 5 μg and 10 μg vaccinated groups induced the effective protection in goats, reduced mean FEC by 71.8% and 68.6%, and mean worm burdens by 69.8% and 61.6%, respectively, compared to the adjuvant control. In addition, we detected that the serum antibody responses to the Con A-purified proteins were dominated by the IgG subtype, but the mucosal antibody responses were not detected. These data demonstrate Con A-purified proteins induced effective immunoprotection in goats, and underline their significance for controlling this widespread parasite.

## 1. Introduction

Parasitic nematodes, particularly gastrointestinal nematodes, are important pathogens that infect hundreds of millions of sheep, goats, and cattle worldwide [1,2,3,4], causing major economic losses estimated at tens of billions of dollars per annum [5]. Current control of these nematodiases relies heavily on the use of chemical drugs, however repeated treatments have led to widespread anthelmintic resistance in nematode populations [6,7,8]. An alternative control method, such as vaccination [9,10], is urgently needed. However, most research on developing anti-nematode native or recombinant vaccines for use in animals has not succeeded [11,12]. To date, only one effective vaccine (Barbervax, https://barbervax.com/ (accessed on 1 October 2021) which has been commercially available in Australia since 2014, protects livestock animals against an economically important gastrointestinal nematode of ruminant animals—*Haemonchus contortus* (the barber’s pole worm) [13].

Barbervax is a native protein vaccine that contains a mixture of membrane glycoproteins, such as H11 and H-gal-GP (*Haemonchus* galactose containing glycoprotein complex), which are derived from adult *H. contortus* intestine [14,15,16]. The native H11 protein is a dominant vaccine component that is purified by Concanavalin A (Con A, a plant lectin binding to mannoses on glycoproteins) [17]. Previously, substantial immunological trials have tested the protective efficacy of the Con A-purified H11 protein. This antigen has been shown to be of particular value in stimulating high levels of antibody-mediated protection, and conferring high levels (70–95%) of immune protection in sheep breeds [16,18,19]. For example, immunization of Clun Forest sheep with 100 μg of Con A-enriched native proteins resulted in more than 95% protection compared with the adjuvant controls [20]. Immunization of Dorset Horn sheep with 1.18 mg of Con A-purified proteins resulted in a 98.5% reduction in the total worm egg output and a 86% reduction in average worm numbers [21]. So far, the majority of the immunized animal tests by the Con A-purified proteins have been reported in different sheep breeds, however, experimental data on the goat breeds remains largely lacking.

Here, we assessed the immunoprotective effect of Con A-purified proteins for controlling the *H. contortus* in Boer goats. This work investigates the parasite native antigen as an alternative approach for preventing this pathogenic *H. contortus*.

## 2. Materials and Methods

### 2.1. Experimental Animals 

Four-month-old Boer goats were purchased from the Hubei Academy of Agricultural Sciences (Wuhan, China). All goats were treated with a single dose of levamisole (7.5 mg/kg, Longyu Biotechnology, Wuhan, China) and three doses of sulfachloropyrazine sodium (12 mg/kg, Youxin Biotechnology, Binzhou, China) prior to purchasing the animals. Two weeks after the anthelmintic treatment, all goats were monitored for parasite eggs for three times by the salt flotation method, and no parasite eggs could be detected. All goats were then transferred into the Laboratory Animal Center of Huazhong Agricultural University. They were fed with alfalfa pellets and ample water daily, and adapted to the indoor environment for one month before starting the trials. The experimental protocol was approved by the Animal Research Ethics Committee of Huazhong Agricultural University (permit HZAUGO-2020-0013).

### 2.2. Parasites

The *H. contortus* strain used in the experiment was originally obtained from the goats in the Hubei Academy of Agricultural Sciences and maintained in the goats at the Laboratory Animal Center of Huazhong Agricultural University. Adult worms were collected from the abomasa of the goats. Briefly, adult *H. contortus* were identified, collected, and then thoroughly washed by sterile phosphate buffered saline (PBS). All adult worms were kept in liquid nitrogen until required. Infective third-stage larvae (L3s) were obtained from coproculture, isolated, and maintained in a 15 °C incubator.

### 2.3. Antigen Preparation

Native proteins were isolated from adult *H. contortus* by Con A lectin-agarose (Vector laboratories, Newark, CA, USA) as reported previously [22]. In brief, 800 adult worms were homogenized in a tris-buffered saline buffer containing 1.0% *v*/*v* Triton X-100 for 30 min in a glass homogenizer. The homogenate was centrifuged at 2500 g for 20 min. The supernatant was filtered (0.22 μm) and purified by Con A lectin-agarose. The bound samples were extensively rinsed with a 0.25% *v*/*v* Triton X-100 buffer, followed by the elution of proteins in a buffer containing 200 mM methyl-α-D-mannopyranoside (Sigma-Aldrich, St. Louis, MO, USA) and 200 mM methyl-α-D-glucopyranoside (Sigma-Aldrich, St. Louis, MO, USA). The protein concentration was detected using a BCA kit (Beyotime, Shanghai, China). 

### 2.4. Vaccination Trials

**Figure 1:**
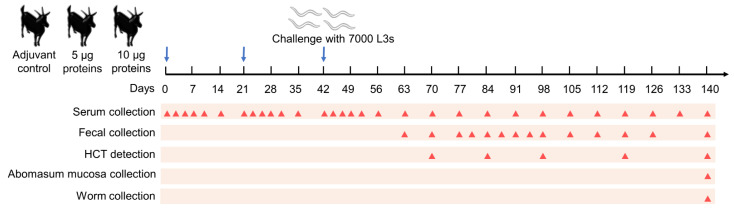
Animal experiment design. Three groups of five goats each were vaccinated with adjuvant alone (control), 5 μg, and 10 μg Con A-purified proteins mixed in adjuvant. All goats were injected subcutaneously thrice (days 0, 21, and 42; blue arrows) at three-week intervals and then challenged with 7000 third-stage larvae (L3s) on day 42. The time points for serum collection (30 times), fecal collection (14 times), hematocrit (HCT) detection (five times), abomasum mucosa collection (once), and worm collection (once) are indicated by red triangles.

According to sex and body weight, fifteen goats were equally divided into three groups: a 5 μg experimental group immunized with a 5 μg/dose of Con A-purified proteins mixed in Quil A adjuvant (InvivoGen, San Diego, CA, USA); a 10 μg experimental group that was immunized with a 10 μg/dose of Con A-purified proteins mixed in Quil A adjuvant; an adjuvant control group that was immunized with Quil A adjuvant alone. The experimental procedure is summarized in Figure 1. All animals were vaccinated subcutaneously three times at three-week intervals. On the same day, after the third vaccination (day 42), all goats were challenged with 7000 *H. contortus* L3s. On days 37–38 of the trials, two goats in the 5 μg experimental group and one goat in the control group died of heat stress syndrome, so their data were discarded from the calculations.

### 2.5. Parasitology

Fecal egg count (FEC) was monitored weekly from day 63 of the trials (day 21 post-challenge infection) to necropsy (day 140) when the worm numbers were counted (Figure 1). FEC (eggs per gram, EPG) was detected according to the McMaster counting method [23]. Briefly, 2 g of feces was homogenized in 58 mL of saturated salt solution. The mixture was filtered and collected in a new beaker. Then the filtrate was mixed and pipetted into two chambers of the McMaster slide, followed by standing for 5 min. The number of eggs was counted under two etched areas on the slide using a microscope, and the egg number multiplied by 100 is the eggs per gram of feces. Each fecal sample was tested in triplicate. The mean cumulative FEC of each group was calculated and the egg reduction rate (%) was analyzed by a calculation [(the mean FEC value for control − mean FEC value for test group) ÷ mean FEC value for control × 100%]. On day 140 of the animal experiment, all goats were euthanized and the numbers of adult worms were collected and counted in the goat’s abomasa.

### 2.6. Antibody Levels

The antibody responses in serum and abomasum mucosa to Con A-purified proteins were determined by enzyme-linked immunosorbent assay (ELISA) as established previously [24]. Briefly, proteins were diluted to 3 μg/mL in carbonate buffer (50 mM, pH 9.6) and coated on microtiter plates (Thermo Fisher Scientific, Waltham, MA, USA) overnight (4 °C). After washing with 1 × PBST, the plates were blocked with 1% BSA in PBS (37 °C, 2 h). Then, 100 μL of the diluted serum (1/2000) and abomasum mucosa (homogenate solution) were added to each well (37 °C, 1 h). The plates were added to diluted microplate labeled secondary antibodies 1/500 IgG (Beyotime, Shanghai, China), 1/5000 IgA (Bio-Rad, Hercules, CA, USA), or 1/5000 IgM (Bethyl, Montgomery, TX, USA) (37 °C, 40 min). Finally, the substrate tetramethylbenzidine was incubated for visualization and the optical density (OD) values were measured at 450 nm.

### 2.7. Hematocrit (HCT)

Each goat’s venous blood (1 mL) was collected using a sodium citrate anticoagulation tube. The degree of anemia in individual goats was detected using a BC2600 hematology analyzer (Mindray, Shenzhen, China) according to the instructions of the reagent dealer.

### 2.8. Statistical Analysis

Statistical analysis was performed using GraphPad Prism 8.0 software (GraphPad Software, San Diego, CA, USA). One-way ANOVA was applied to compare the statistical differences between groups. The * *p* < 0.05, ** *p* < 0.01, *** *p* < 0.001, and **** *p* < 0.0001 indicate the degree of significance, as shown in respective graphs.

## 3. Results

### 3.1. Fecal Egg Count (FEC)

**Figure 2:**
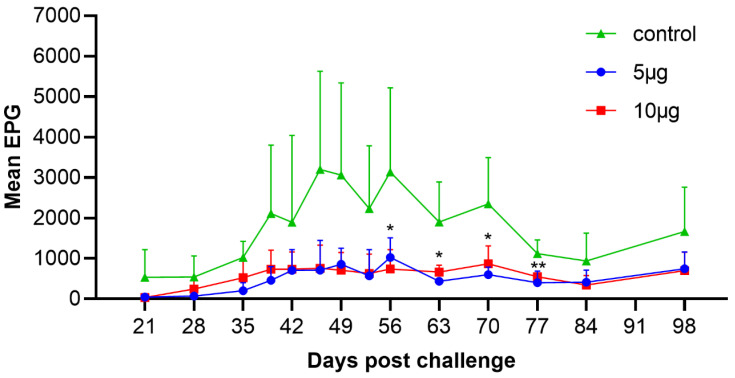
Dynamic range of fecal eggs per gram (EPG) in the trials. Goats vaccinated with adjuvant alone were served as the controls for those vaccinated with 5 μg and 10 μg protein groups. Mean group EPG (mean ± SD; adjuvant control, *n* = 4; 5 μg group, *n* = 3; 10 μg group, *n* = 5) was monitored at 14 time points (days 21–98 post-challenge infection). Statistical significance was determined using the one-way ANOVA, * *p* < 0.05 and ** *p* < 0.01.

We assessed the dynamic range of mean EPG values on days 21–98 post-challenge infection. The results showed that goats began to excrete *H. contortus* eggs around day 21 post-infection. The mean EPG of the control animals continuously increased, and reached the peak on day 46 (3200 ± 2428), whereas the mean EPG of both 5 μg and 10 μg vaccinated groups maintained at low (<1000) level until the end of the trials. Compared with the adjuvant control, the mean cumulative FEC of 5 μg and 10 μg vaccinated groups showed egg reduction in 71.8% and 68.6%, respectively (Figure 2; Table 1). However, no significance was observed in mean cumulative FEC between the 5 μg and the 10 μg experimental groups.

### 3.2. Worm Burdens

We further tested the adult worm counts in the two vaccinated groups and one control group. Compared with the control group, goats immunized with 5 μg and 10 μg Con A-purified proteins had significantly (*p* < 0.05) fewer female, male, and total worm numbers at necropsy (Table 2). The total worm numbers of the 5 μg and 10 μg experimental groups were reduced by 69.8% and 61.6%, respectively, compared to the control group (Table 2). Nevertheless, the worm burdens were not statistically significant between the 5 μg and 10 μg experimental groups.

### 3.3. Antibody Responses to Con A-Purified Proteins 

**Figure 3:**
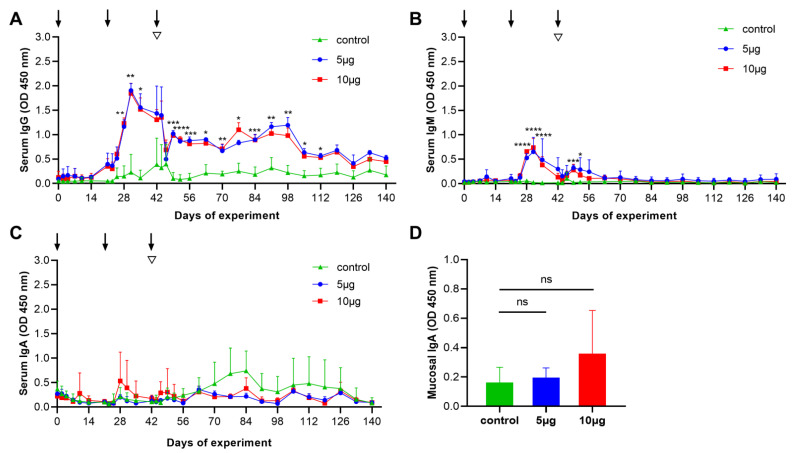
Serum and mucosal antibody responses of goats vaccinated with adjuvant alone (control), 5 μg, and 10 μg Con A-purified proteins, were measured by enzyme-linked immunosorbent assay (ELISA). (**A**–**C**) Dynamics of serum IgG (**A**), IgM (**B**) and IgA (**C**) antibody responses to Con A-purified proteins were detected in three experimental groups at 30 time points (see Figure 1). Each data (OD 450 nm) denoted the mean antibody titers (mean ± SD; adjuvant control, *n* = 4; 5 μg group, *n* = 3; 10 μg group, *n* = 5). The three time points (days 0, 21, and 42) of the immunizations are indicated by arrows, and the time point (day 42) of challenge with 7000 third-stage larvae (L3s) is indicated by inverted triangles. (**D**) Each data (OD 450 nm) denoted the mean mucosal IgA antibody titers at the trials (mean ± SD; adjuvant control, *n* = 4; 5 μg group, *n* = 3; 10 μg group, *n* = 5). Statistical significance was determined using the one-way ANOVA, * *p* < 0.05, ** *p* < 0.01, *** *p* < 0.001, **** *p* < 0.0001, and ns (nonsignificant).

To assess the antibody responses in the vaccinated goats, the serum IgG, IgM, and IgA antibody responses to the Con A-purified proteins were measured by indirect ELISA. Specific serum IgG antibody responses in vaccinated groups (5 μg and 10 μg groups) were distinctly detected on day 28 and then increased swiftly to reach the peak value on day 31 of the trials. Serum IgG antibody responses were maintained at significantly higher levels (*p* < 0.05) from days 28 to 112 in comparison to that of the control (Figure 3A). In addition, specific IgM levels were significantly (*p* < 0.0001) higher in titers detected from days 28 to 35 compared with the control (Figure 3B), whereas there were no statistical changes detected in serum IgA in any experimental group (Figure 3C). Furthermore, we also determined the mucosal IgA antibody responses of the experimental goat’s abomasa. However, specific mucosal IgA was not detectable in the signals from any animal group (Figure 3D). These data, taken together, suggest that Con A-purified proteins primarily induced the serum IgG subtype in the goats.

### 3.4. Degree of Anemia

Given that infection of *H. contortus* could cause animal anemia, we determined the degree of anemia of each goat based on the HCT assay. The result showed that there was no significant difference between the three experimental groups, although one goat in the control group developed anemia on day 56 after the challenge.

## 4. Discussion

Con A-purified proteins have been shown to confer high levels (70–95%) of immune protection in most sheep breeds [17,21,25], but study of the immunoprotection efficacy of this native antigen remains largely lacking in goat breeds. In the present study, we evaluated Con A-purified proteins as the vaccine potential for controlling *H. contortus* infection in goats. We demonstrated that low-dose Con A-purified proteins could induce effective immune protection (reducing the number of eggs > 69% and the number of worms > 62%) and high levels of specific IgG antibodies in the 140-day trials. 

Con A-purified native proteins have a complex extraction process and low purification concentration, and production depends on animal donors. Therefore, the vaccination doses and schedule used in animals should consider economic costs. Previously, immunization with Con A-purified proteins in sheep was tested in high doses (>100 μg) and most studies were assessed in short-term (about 60 days) experiments [17,20,25]. By contrast, our results showed the low-dose Con A-purified proteins could induce long-term immunoprotective effects in goats. Nevertheless, our data showed that the immunoprotection effect of Con A-purified proteins in goats is lower than those in sheep breeds [20,21]. Due to different immunization doses, the different immune responses between goats and sheep are unclear, so further work would be required to clarify this difference under the same immunization protocol.

Previous studies have indicated that Barbervax can induce effective immunoprotection in different goat breeds. In a trial performed in Brazil, female Saanen and Anglo Nubian goats, vaccinated with 5 μg Barbervax, resulted in 57.4% and 69.8% egg reduction in Saanen and Anglo Nubian goats, respectively [26]. Subsequent experiments showed that this vaccine could provide >65% immune protection in pregnant goats [27]. In another trial performed in Switzerland, goats aged from 2 to 5 months were immunized with 5 μg Barbervax in four-week or six-week intervals in a grazing experiment. Mean numbers of adult worms deriving from experimental infections were significantly reduced by 89% in the 4-week interval immunization group, while the 6-week interval immunization group was reduced by 47%, compared with the controls [28]. Although the Barbervax component contains Con A-purified H11 molecules and PNA (Peanut agglutinin)-purified H-gal-GP complex [29,30,31,32], our study is consistent with the immunoprotection effect of Barbervax. We believe that Con A-purified proteins do possess an excellent immunogenicity that could be employed in all goat breeds. 

We showed that the antibody responses to the Con A-purified proteins (5 μg and 10 μg groups) were dominated by the IgG subtype. In accord, previous studies have demonstrated that native proteins (e.g., H11 antigen) can induce the prominent serum IgG antibody responses [21]. We observed that the highest antibody titers were observed on day 28 of the trials (day 7 after the second immunization), which has a significant difference (*p* < 0.05) from days 28–112 of the trials, compared with the control. Serum IgG antibody responses have been proven to associate with immune protection, and the protection mechanism of IgG antibodies are hypothesized to disrupt the intestinal nutrient absorption in this parasite [33]. However, at the end of the experiment, we observed that the IgG antibody levels in the vaccinated groups had dropped to the points (days 112–140) where they were not significantly different from the control group, suggesting that the IgG antibody responses induced by the Con A-purified proteins may be not durable. The duration of antigen delivery will be important for stimulating a durable immune response and an effective protection in animals [34,35]. With the development of biotechnology, there are many nanoparticle adjuvant systems, such as VLPS, ISCOM, and AS01 [36,37,38,39,40], that can encapsulate antigen proteins to release slowly at the injection site [41,42], which could help the low-dose antigen to improve immune efficacy and persistence. Accordingly, building up a suitable nanomaterial system to encapsulate *H. contortus* native antigen is necessary for achieving the long-term immunoprotection against this parasite infection in the future.

In addition to the IgG antibody, we also detected that serum IgM antibody responses were produced in the early stages (days 28–35 of the trials). Compared with the control group, IgM antibody responses in two vaccinated groups briefly increased. However, the IgM antibody levels dropped to the lowest level when all animals were challenged with the *H. contortus* L3s, indicating that this antibody might not play an immune protective role in against *H. contortus* infection [43]. Furthermore, our study showed that the mucosal IgA antibody responses of the two vaccinated groups were higher than that of the control, but there was no statistical difference, which indicates that immunization with Con A-purified proteins is impossible to induce mucosal immunity in the goat’s abomasum.

The immune protection tests of the Con A-purified proteins demonstrated *H. contortus* native proteins could serve as an alternative for the control of *H. contortus* infection in goats. However, our study was used simply to determine whether Con A-purified proteins could be of any use for the goats. The vaccination procedure of the native antigen used in the current study may not be optimal for inducing a high level of immune protection in grazing animals. To realize a balanced and long-term *H. contortus* control, further testing with optimal vaccinated conditions, including a better immunization dose and interval in animals, is necessary.

## 5. Conclusions

In the present study, we investigated the immunoprotective effect of low-dose Con A-purified proteins for controlling *H. contortus* infection in goats. We showed that low-dose Con A-purified proteins can induce effective immune protection and high levels of specific IgG antibodies in the 140-day trials. Our work, therefore, demonstrates the immunoprotective effect of Con A-purified proteins in goats and will facilitate the vaccine development against this widespread parasitic nematode. 

## Figures and Tables

**Table 1:** Fecal egg count (FEC) from challenged goats in the trials. Fecal samples were collected from individual goats at 14 time points (see Figure 1), and the numbers of *Haemonchus contortus* eggs per gram were counted. Mean cumulative FEC (with standard deviations, SD) of individual groups was calculated.

Groups	Mean FEC	SD	Reduction (%) ^a^
Control 5 μg	25,587.3 7211.7	14,637.8 3875.8	– 71.8 *
10 μg	8039.8	4189.2	68.6 *

^a^ The reduction (%) = [(the mean FEC value for control − mean FEC value for test group) ÷ mean FEC value for control × 100%]. Statistical significance was determined using one-way ANOVA, * *p* < 0.05.

**Table 2:** Worm numbers from challenged goats in the trials. At the end of the experiment (day 140), the worm numbers in the goat’s abomasa were counted. Mean worm numbers (with standard deviations, SD) of individual groups were counted.

Groups	Female			Male			Total		
	Mean	SD	Reduction (%) ^a^	Mean	SD	Reduction (%) ^a^	Mean	SD	Reduction (%) ^a^
Control 5 μg	133.3 43.3	70.1 18.9	– 67.5 *	99.2 27	40.6 17.1	– 72.8 *	232.5 70.3	109.4 35	– 69.8 *
10 μg	47	15.6	64.7 *	42.2	25.8	57.5 *	89.2	41.1	61.6 *

^a^ The reduction (%) = [(the mean worm burden for control − mean worm burden for test group) ÷ mean worm burden for control × 100%]. Statistical significance was determined using one-way ANOVA, * *p* < 0.05.

## Data Availability

Not applicable.
